# Does depression in Alzheimer's disease show evidence of differential gene expression in neurons or microglia compared to Alzheimer's disease without depression?

**DOI:** 10.1002/alz70857_105622

**Published:** 2025-12-25

**Authors:** Lindsey I Sinclair, Mizuki Morisaki, Peter P Henley, Emma Dempster, Clive G Ballard, Jordan T Lin, Seth Love

**Affiliations:** ^1^ University of Bristol, Bristol, United Kingdom; ^2^ Cardiff university, Cardiff, Cardiff, United Kingdom; ^3^ University of Bristol, Bristol, UK, United Kingdom; ^4^ University of Exeter, Exeter, United Kingdom; ^5^ University of Bristol, Bristol, Horfield, United Kingdom

## Abstract

**Background:**

Depression in individuals with Alzheimer's disease (AD) is common, distressing, does not respond to currently available antidepressants and is inadequately understood. We aimed to identify patterns of differential gene expression in depression in AD compared to AD without depression, including the influence of depression genetic risk on gene expression.

**Method:**

We identified individuals with AD +depression (*n* = 95) and AD without depression (*n* = 25) with tissue available from UK brain banks. Exclusion criteria included; other disorders known to affect cognition; major psychiatric disorder; significant depression prior to AD diagnosis; and a primary neuropathological diagnosis of a non‐AD dementia. Depression was defined as a clinical diagnosis or GDS/CESD >7. Fill in genotyping was performed using UKBiobank axiom array and imputed using the Michigan Imputation server. PRS‐depression was calculated using LDpred2. In the AD +depression group only the top (*n* = 29) and bottom tertile (*n* = 30) of PRS‐depression were measured using microarray.

Tissue from the superior frontal gyrus (SFG) and the anterior insula (AIns) was gently homogenised, FACS sorted into neuronal and microglial fractions and gene expression measured using a ClariomS Pico microarray, prior to analysis in TAC console (ThermoFisher). Covariates included age, sex, braak stage, postmortem delay, and RIN. Our a‐priori power analysis found 30 per group gives 90% power to detect a 2‐fold gene expression change. Our primary outcome was gene expression in neurons comparing AD +depression to AD no depression.

**Result:**

Following quality control, microarray data was available for AIns microglia (*n* = 53), AIns neurons (*n* = 61), STG microglia (*n* = 61) and STG neurons (*n* = 65). No individual genes had an FDR adjusted p value <0.05. Gene set enrichment analysis (GSEA) in R identified significantly upregulated and downregulated pathways in both brain regions and cell types, as well as in most of the PRS comparisons in the secondary analysis. More pathways were differentially expressed in microglia than in neurons e.g. in the AIC microglia, 8 pathways were significantly upregulated and 25 downregulated in AD+ depression vs AD no depression.

**Conclusion:**

Although no individual gene reached significance, by using GSEA we have identified pathways which may contribute to the development of depression in AD, particularly in microglia.

